# An LLM driven dataset on the spatiotemporal distributions of street and neighborhood crime in China

**DOI:** 10.1038/s41597-025-04757-8

**Published:** 2025-03-20

**Authors:** Yan Zhang, Mei-Po Kwan, Libo Fang

**Affiliations:** 1 Department of Geography and Resource Management, The Chinese University of Hong Kong, Shatin, Hong Kong SAR; 2 Institute of Space and Earth Information Science, The Chinese University of Hong Kong, Shatin, Hong Kong SAR; 3 Hunan Architectural Design Institute Group Co., Ltd, Changsha, China

**Keywords:** Geography, Society, Developing world

## Abstract

Crime is a significant social, economic, and legal issue. This research presents an open-access spatiotemporal repository of street and neighborhood crime data, comprising approximately one million records of crimes in China, with specific geographic coordinates (latitude and longitude) and timestamps for each incident. The dataset is based on publicly available law court judgment documents. Artificial intelligence (AI) technologies are employed to extract crime events at the neighborhood or even building level from vast amounts of unstructured judicial text. This dataset enables more precise spatial analysis of crime incidents, offering valuable insights across interdisciplinary fields such as economics, sociology, and geography. It contributes significantly to the achievement of the United Nations Sustainable Development Goals (SDGs), particularly in fostering sustainable cities and communities, and plays a crucial role in advancing efforts to reduce all forms of violence and related mortality rates.

## Background & Summary

Street crime and neighborhood crime represent distinct spatial patterns of criminal behavior, with the former occurring in public spaces such as streets and squares, while the latter predominantly manifests within residential areas and communities. These forms of crime, primarily concentrated in densely populated urban areas, pose significant threats to human life, property security, and psychological well-being^1^. The United Nations Sustainable Development Goals (SDGs) emphasize the importance of addressing these issues through SDG 11 (Sustainable Cities and Communities), which calls for inclusive, safe, resilient, and sustainable urban settlements, and SDG 16 (Peace, Justice, and Strong Institutions), which aims to combat organized crime and reduce all forms of violence and related mortality rates. Consequently, the accurate identification of street and neighborhood crime locations holds dual significance. For the general public, such identification enhances risk awareness and enables informed decisions about one’s movement patterns in high-crime areas. For governmental authorities, comprehensive spatiotemporal crime data facilitates optimal police resource allocation and targeted interventions in crime-prone communities. Given the immediate relevance of street and neighborhood crime to urban residents, considerable research has been conducted in this domain. Studies examining street crime have thoroughly investigated the relationship between built environment characteristics and crime risk. Similarly, neighborhood crime research has explored the relationships between crime and community quality, property values, perceived safety, and socioeconomic status^2–4^. Research findings consistently indicate that street and neighborhood crimes exhibit non-random distributions, often displaying distinct spatiotemporal clustering characteristics that form crime hotspots^5–9^. The emergence of these hotspots is intrinsically linked to various urban factors, including population density, land use patterns, population mobility, and architectural configuration^10–14^.

Comprehensive and reliable street and neighborhood crime data entail three fundamental attributes: temporal information (when the crime occurred), spatial information (where the crime took place), and contextual information (the nature of the criminal activity). Compared with other forms of criminal activity such as economic crimes, street and neighborhood crime has precise spatial granularity, often pinpointing specific streets or buildings. This spatiotemporal information is instrumental in identifying crime hotspots and analyzing their evolutionary patterns. Western countries, particularly the United States and the United Kingdom, have established sophisticated crime dataset infrastructures. Cities such as Chicago (data.cityofchicago.org), New York, Los Angeles, and London (data.police.uk) maintain publicly accessible crime data interfaces. These rich data sources have facilitated a wide range of interdisciplinary research and are helpful in protecting the lives and property of residents.

However, large-scale, reliable, and publicly accessible street crime data of mainland China remains limited. This limitation may be attributed to varying levels of economic development, social environment, and governmental transparency. This data deficiency has resulted in a significant knowledge gap regarding the spatiotemporal characteristics of street and neighborhood crime in developing countries and their relationships with socioeconomic and built environment factors^15,16^. China, as the world’s largest developing country, has undergone unprecedented urbanization over the past two decades, with urbanization rates increasing from 36.22% in 2000 to 66.16% in 2023. This rapid urbanization has precipitated substantial spatial and social restructuring of urban areas, potentially contributing to increased crime incidents. Given the importance of crime research in developing countries, several studies have addressed this research gap. However, these studies mainly rely on restricted-access data from public security institutions, limiting computational analysis within internal networks and precluding public data sharing^17–20^. This restricted access hinders comprehensive cross-validation and comparative analysis of findings across different cities.

How can we obtain large-scale crime spatiotemporal data in China while maintaining reasonable accuracy and maximizing privacy protection? To address this challenge, we present a dataset comprising approximately 1 million street and neighborhood crime records, encompassing 31 provincial-level administrative regions, 222 city-level divisions, and 548 county(district)-level jurisdictions across mainland China. The dataset exhibits extensive potential applications across multiple domains. In China’s administrative systems, provinces represent the highest level of administration, followed by city-level, and then counties or urban districts. These administrative units form a nested structure where provinces contain multiple cities, and cities contain multiple counties/districts. This three-tiered system is consistently used throughout our dataset to ensure standardized spatial reference and analysis.

It enables the assessment of bidirectional relationships between criminal activities and various urban factors, including built environment characteristics, real estate values, urbanization levels, and population mobility patterns. The dataset facilitates sophisticated spatiotemporal distribution and hotspot analyses, as well as difference-in-differences (DID) analyses of policy interventions. Furthermore, being grounded in real-world scenarios, it provides valuable support for evaluating existing police station deployment, patrol route designs and crisis management simulations. By making this dataset publicly accessible, we aim to democratize data access and provide essential evidence-based decision-making resources for researchers, policymakers, and the general public. Moreover, this research shows how to obtain the high-quality spatiotemporal dataset based on the loosely structured open-source information through an AI for Science method. This contribution represents a significant advancement in crime research infrastructure, particularly for developing nations.

## Methods

The collection of large-scale spatiotemporal crime data in China requires reliable and accurate sources while safeguarding privacy concerns. The China Judgments Online platform (https://wenshu.court.gov.cn/), operated by the Supreme People’s Court of China, offers a potential solution. It serves as a unified national repository for court judgments or decisions^21,22^. As part of the judicial transparency reform, the platform provides over 100 million nationwide, de-identified judicial documents of criminal cases.

Different crime data sources have their distinct characteristics. Court judgment document data features highly standardized formats and structures, with information verified through judicial procedures. It contains rich details about time, location, and case circumstances, maintaining national consistency and public accessibility. In contrast to it, police record data encompasses all reported incidents, offering real-time information and high classification accuracy with detailed initial investigation information. But it is typically restricted to internal use, with inconsistent recording standards across regions and limited public access. Additional sources include insurance company data and social media news data, each facing various challenges such as limited case coverage and information authenticity issues. Therefore, this research’s dataset contributes to a more comprehensive understanding of criminal activities and patterns by providing judicially reviewed, detailed, and standardized crime case information.

**Fig. 1:**
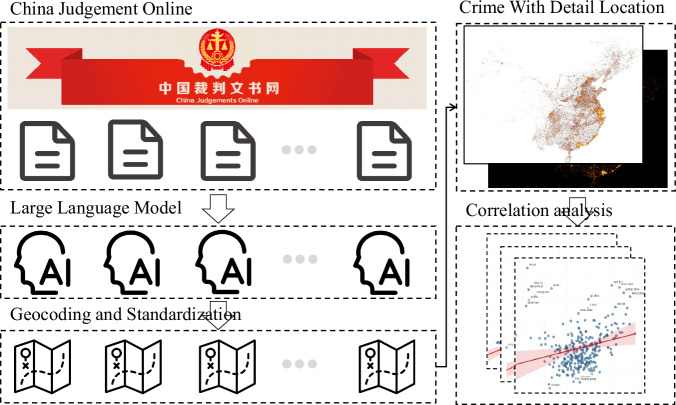
Schematic diagram of data processing workflow.

By using keywords such as robbery, snatching, and theft, we retrieved more than 2 million court decision documents related to street crimes and neighborhood crimes. The documents contain information about the time and location of crimes, stolen items, and specific sentencing details with unformatted text, while data analysis mostly requires structured panel data. Extracting structured data from large volumes of unstructured text is undoubtedly challenging. However, advances in big data and artificial intelligence technologies, particularly large language models (LLMs) like ChatGPT, have provided us with more effective solutions^23,24^. Subsequently, we utilized Baidu Maps API’s geocoding service to encode geographical coordinates based on textual crime locations. The specific workflow is illustrated in Fig. 1.

### Dataset construction framework

#### Standardization of unstructured text using large language model

In this study, we extracted structured data from the China Judgments Online platform. Using keywords such as robbery, snatching, and theft, we retrieved over 2 million court decision documents related to street and neighborhood crimes. These documents formed a substantial corpus containing approximately 2 billion tokens for text processing. Given the massive scale of the dataset, manual identification of critical information (such as addresses, timestamps, and other relevant details) would take too much work. Some research has demonstrated that LLMs can be effectively applied to named entity recognition (NER) tasks^25^. There are numerous existing LLMs provided by companies such as OpenAI, Anthropic, Alibaba, Google, and Baidu, offering commercial application programming interfaces (API). Based on model output quality and API pricing, we selected the Gemini-1.5-Flash-Latest model API from Google. The prompt used for extraction is as follows:
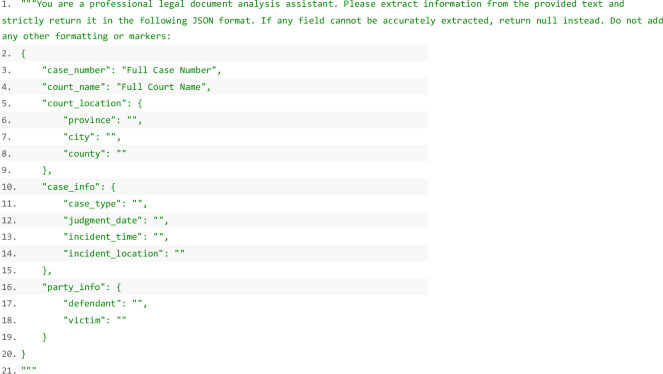


#### Address geocoding based on baidu maps API

The process of converting a textual description of crime location into specific geographical coordinates (with latitude and longitude) is known as geocoding, a common task in geography. Geocoding involves converting structured address data (e.g., “129 Luoyu Road, Hongshan District, Wuhan”) into corresponding geographic coordinates. We utilized the Baidu Maps API, one of China’s largest online mapping platforms, to encode text-based address descriptions. This API has been widely applied in fields such as economics and geography and has demonstrated high accuracy^26^. Additionally, for cases involving multiple crime locations by the same judgment, we primarily extracted the first one as the main crime location for the next step analysis.

#### Standardization of crime time calculation

To convert the date and time information extracted from Chinese text by the LLM into a standardized date-time format, we designed a parsing method based on regular expressions. The core logic of this method consists of two parts: the first part matches the date components, including year (YYYY), month (MM), and day (DD); the second part matches the time components, which include specific hour values or time periods (e.g., “morning,” “afternoon”). When a specific hour is matched, the system directly converts it into a 24-hour format. For time periods such as “noon” or “evening,” the system uses predefined mappings to convert them into corresponding hour values (e.g., “noon” is converted to 12:00, “evening” is converted to 20:00). For example, the input text “2024年1月5日 上午10时“ will be successfully matched, extracting the year (2024), month (1), day (5), and hour (10) using regular expressions. The system then generates the standardized time “2024-01-05 10:00,” which complies with the ISO 8601 standard, facilitating cross-disciplinary and cross-domain data processing and analysis. In cases where no explicit time information is provided, the system returns “NaT” (Not a Time), indicating that the parsed time information cannot be parsed into a standard time format.

## Data Records

**Fig. 2:**
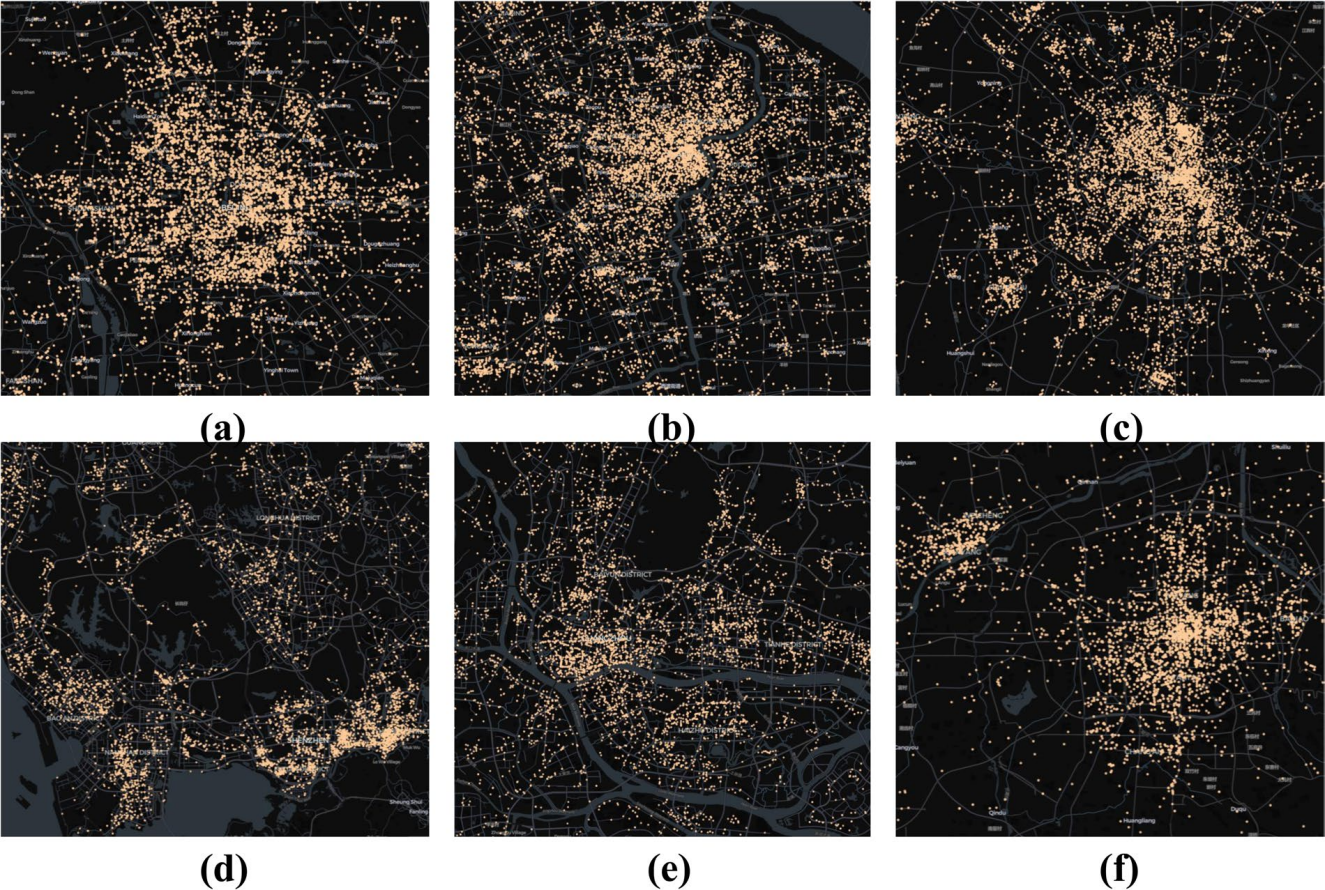
The spatial distribution of all recorded crime incidents across major urban districts of six selected metropolitan areas.

The dataset, totaling approximately 7 GB, encompasses a range of key fields about each crime case. These fields include the unique case identifier (case_number), the type of case (case_type), the name of the court issuing the judgment (court_name), and the city of the court (city). Additionally, it contains detailed information about the crime location, including a textual description (incident_location) and its geographic details, such as province, city, and county, labeled as incident_province, incident_city, and incident_country. Temporal information is captured in both the textual format (incident_time) and a standardized timestamp (formatted_datetime), alongside the judgment date (judgment_date). Geospatial data is provided through the longitude and latitude coordinates (longitude and latitude). The dataset also includes information about the victim (victim) and defendant (defendant), detailed crime descriptions (detail), and the original judicial documents (judgment). The dataset is publicly accessible on Figshare^27^ and the field descriptors can be found at the Supplementary Table 1. This table provides a comprehensive overview of the criminal case dataset structure, including field specifications, data types, examples, and usage guidelines. Personal information is partially anonymized in compliance with privacy regulations. Figure 2 shows the crime distribution across six different cities. The incidents cluster in densely populated areas, as shown in panels A-F for Beijing, Shanghai, Chengdu, Shenzhen, Guangzhou, and Xi’an.

## Technical Validation

### The spatiotemporal distribution of the dataset

**Fig. 3:**
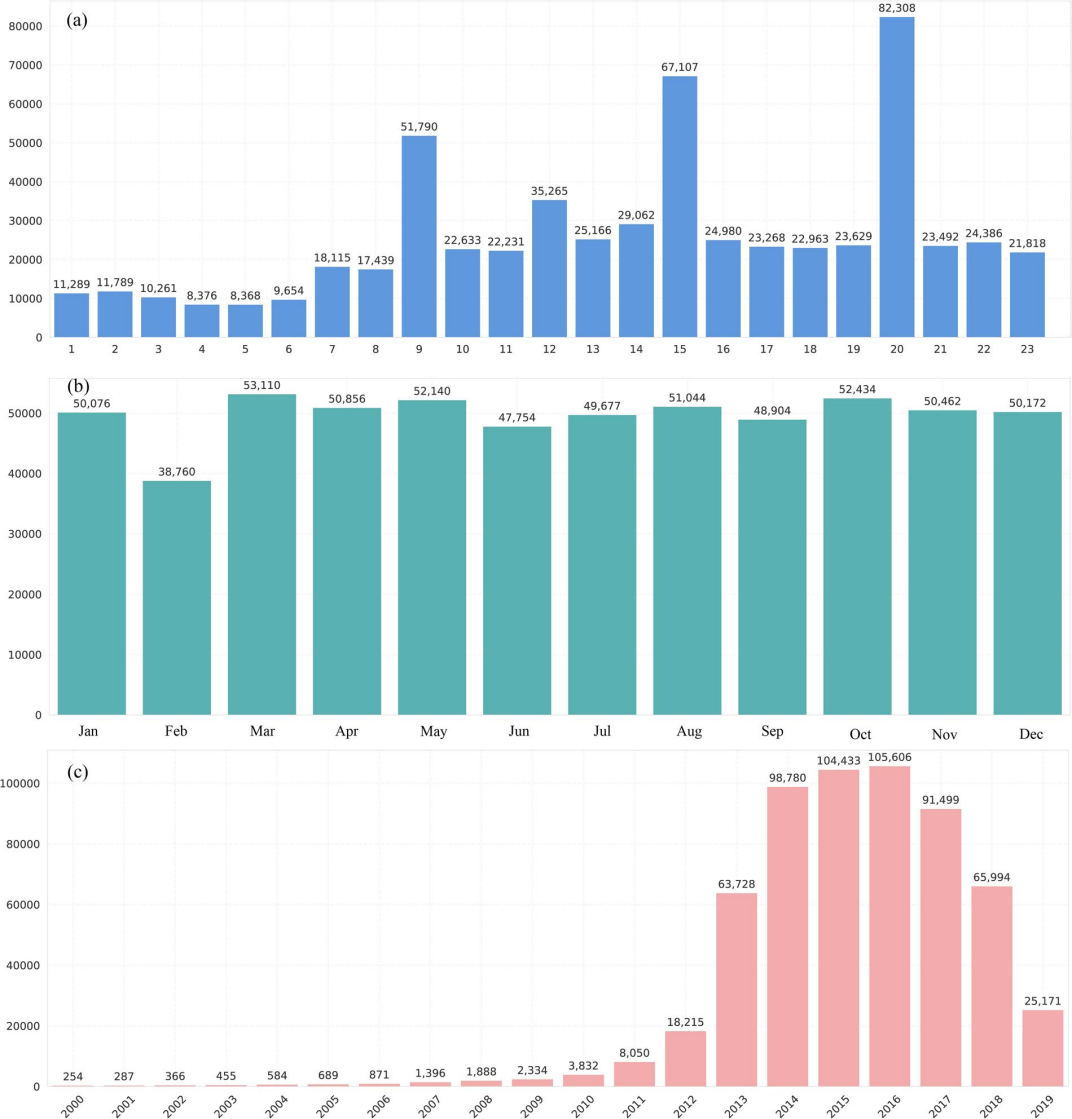
Temporal distribution of all recorded criminal cases. Panels (**a,b**), and (**c**) represent the distribution of cases across a 24-hour period, monthly distribution, and yearly distribution, respectively.

To better understand the spatiotemporal distribution of the dataset, we constructed Fig. 3, where panels (a), (b), and (c) represent the distribution of cases over a 24-hour period, by month, and by year, respectively. This figure shows that February experiences the fewest street and neighborhood crimes, likely due to the Chinese New Year, which typically occurs in February and may lead to a decrease in criminal activity. We also observed higher crime rates at 9:00 AM, 12:00 PM, 3:00 PM, and 8:00 PM. This pattern may be attributed to the relatively vague time descriptions used in many case reports, where terms like “morning” or “afternoon” are often used to describe the crime time. Furthermore, the period between 5:00 AM and 6:00 AM sees the least criminal activity. Additionally, due to restrictions on official websites and the declining availability of online judgment documents, the data is primarily concentrated between 2013 and 2019. After 2019, the availability of documents either became limited or was no longer publicly accessible. Another notable reason is the widespread adoption of electronic payment systems, which has reduced cash-carrying behavior and potentially influenced certain types of criminal activities.

**Fig. 4:**
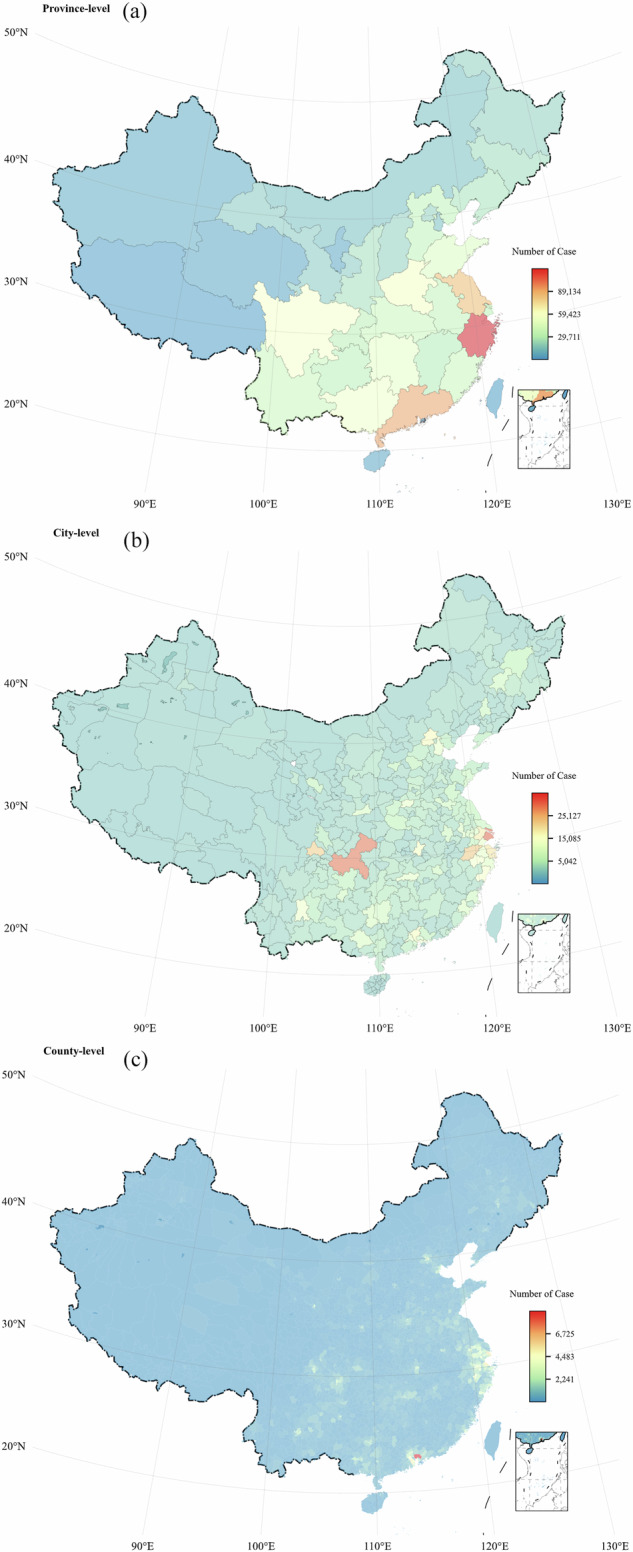
Spatial distribution of all recorded criminal cases. Panels (**a,b**), and (**c**) represent the distribution of crime cases at the province, city, and county level, respectively.

In addition, we plotted the spatial distribution of crimes at the province, city, and county levels (Fig. 4). The dataset covers nearly all regions of mainland China, providing detailed data that makes it the highest-quality publicly available dataset on the spatiotemporal distribution of street and neighborhood crime in China.

### City-Level analysis of crime and socioeconomic correlations

Official statistical yearbooks provide authoritative data, enabling more reliable analysis of relationships between crime patterns and urban socioeconomic characteristics. Given that 2016 represents the peak coverage in our judicial document dataset, we utilized the 2016 statistical yearbook data for the correlation analysis at the city level. We selected six socioeconomic related indicators from it, including the average annual population, gross regional product per capita, average salary of employees, number of registered unemployed persons in urban areas at the end of the year, number of employees in the tertiary industry, and number of employees in the primary industry.

**Fig. 5:**
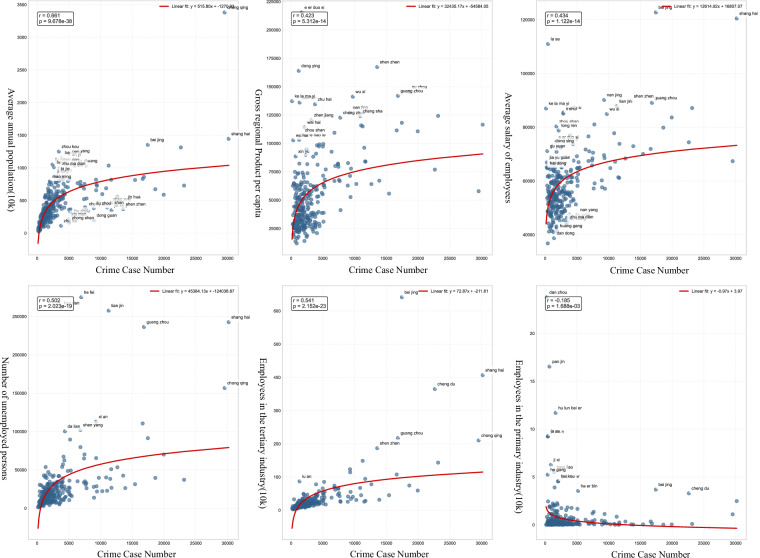
Correlation between urban indicators and the number of crime cases. The figure shows the relationships between six key urban indicators—average annual population, gross regional product per capita, average salary of employees, number of registered unemployed persons, number of employees in the tertiary industry, and number of employees in the primary industry—and the number of crime cases in cities.

As shown in Fig. 5, we plotted the correlations between these six urban indicators and the number of crime cases at the city level. The analysis reveals a strong positive correlation between crime numbers and urban population size as well as the number of registered unemployed persons. However, the relationship between crime and economic development (GDP per capita) and average salary exhibits an inverted U-shape, while the correlation between crime and the tertiary industry workforce shows a positive U-shape.

## Usage Notes

The dataset is publicly available under the Creative Commons Attribution 4.0 International License (CC BY 4.0), allowing for unrestricted access, sharing, and adaptation with appropriate attribution. Researchers, practitioners, and the public can access and download the complete dataset through the Figshare repository (10.6084/m9.figshare.28106939). Users can choose to download either the full dataset or specific components based on their research needs. The data is provided in standard CSV format, ensuring compatibility with common analytical tools and statistical software packages. While the dataset is freely accessible, users are expected to cite the original publication when using the data in their research or applications.

## Supplementary information

Criminal Case Dataset Field Specifications and Descriptors

## Data Availability

The LLM prompt used in this study has been disclosed in the main text. Additionally, readers can access the technical documentation of the API interfaces provided by commercial companies for more information.
