# Supplementary material for: An LLM driven dataset on the spatiotemporal distributions of street and neighborhood crime in China
Source: Sci Data. 2025 Mar 20;12:467. doi: 10.1038/s41597-025-04757-8 (PMC11926219; doi:10.1038/s41597-025-04757-8)
Supplement: Supplementary file 1 — Criminal Case Dataset Field Specifications and Descriptors [file 41597_2025_4757_MOESM1_ESM.docx]

**Supplementary** **Table 1:** Criminal Case Dataset Field Specifications and Descriptors

| **Field Name** | **Description** | **Data Type** | **Example** | **Usage Notes** |
| --- | --- | --- | --- | --- |
| case_number | Unique identifier for each case | String | (2018)粤0902刑初370号 | Format: (Year)Province-Court-CaseType-Number |
| case_type | Classification of the case | String | Criminal offence | Criminal case classification |
| court_name | Name of the presiding court | String | Maonan People's Court | Hierarchical court information |
| geographic_info | Geographic location data | - | - | Multiple fields for spatial analysis |
| - city | Court jurisdiction city | String | Maoming City | Administrative city |
| - latitude | Geographic latitude | Float | 21.679 | Coordinate system: WGS84 |
| - longitude | Geographic longitude | Float | 110.905 | Coordinate system: WGS84 |
| incident_location | Detailed location description | String | Maoming City Guandu Bridge | Text description of crime scene |
| administrative_division | Administrative hierarchy | - | - | Three-level administrative division |
| - incident_province | Province level | String | Guangdong Province | Provincial administrative region |
| - incident_city | City level | String | Maoming City | City administrative region |
| - incident_county | County level | String | Maonan District | County administrative region |
| temporal_info | Temporal information | - | - | Time-related fields |
| - incident_time | Original time description | String | 2017年8月20日17时许 | Raw time information |
| - formatted_datetime | Standardized timestamp | DateTime | 2017-08-20 17:00:00 | ISO format timestamp |
| - judgment_date | Date of court judgment | Date | 2018-08-28 | YYYY-MM-DD format |
| case_parties | Case participants | - | - | Individual information |
| - defendant | Defendant information | String | Ke XX | Name (may be anonymized) |
| - victim | Victim information | String | Huang XX | Name (usually anonymized) |
| case_content | Case details | - | - | Detailed case information |
| - details | Case summary | Text | XXX | Brief case description |
| - judgment | Full judgment document | Text | XXX | Complete court document |
